# Pentagalloylglucose, isolated from the leaf extract of *Anacardium occidentale* L., could elicit rapid and selective cytotoxicity in cancer cells

**DOI:** 10.1186/s12906-020-03075-3

**Published:** 2020-09-21

**Authors:** Bamigboye J. Taiwo, Temidayo D. Popoola, Fanie R. van Heerden, Amos A. Fatokun

**Affiliations:** 1 Department of Pharmaceutical Chemistry, Faculty of Pharmacy, Obafemi Awolowo University, Ile-Ife, Osun State Nigeria; 2 School of Chemistry and Physics, Pietermaritzburg Campus, University of KwaZulu-Natal, Private Bag X01, Scottsville, 3209 South Africa; 3 Centre for Natural Products Discovery (CNPD), School of Pharmacy and Biomolecular Sciences, Liverpool John Moores University, Byrom Street, Liverpool, L3 3AF UK

**Keywords:** *Anacardium occidentale*, Pentagalloyl glucose, Selectivity index, Cytotoxicity

## Abstract

**Background:**

The leaf of *Anacardium occidentale* L. has been a component of many herbal recipes in South-Western Nigeria. The work reported herein, therefore, explored the phytochemical composition of this plant and the potential anti-cancer activity of an isolated chemical constituent.

**Methods:**

Phytochemical methods (including chromatographic analysis) combined with spectroscopic and spectrometric analyses (IR, HRMS and NMR (1D and 2D)) were used to identify chemical constituents. Cytotoxic effects were determined using the MTT viability assay and bright-field imaging. Induction of oxidative stress was determined using the fluorescence-based 2′,7′-dichlorofluorescein diacetate (DCFDA) assay.

**Results:**

For the first time in the plant, **Compound 1** was isolated from the leaf extract and identified as pentagalloylglucose. **Compound 1** was significantly cytotoxic against the cancer cell lines HeLa (human cervical adenocarcinoma cell line) and MRC5-SV2 (human foetal lung cancer cell line), with IC_50_ of 71.45 and 52.24 μg/ml, respectively. The selectivity index (SI) for Compound 1 was 1.61 (IC_50_ against the normal human foetal lung fibroblast cell line MRC-5 was 84.33μg/ml), demonstrating better cancer cell-selectivity compared to doxorubicin with a SI of 1.28. The cytotoxic activity of **Compound 1** in HeLa cells was also rapid, as shown by its concentration- and time-dependent 3 h and 6 h cytotoxicity profiles, an effect not observed with doxorubicin. Generation of reactive oxygen species at high concentrations of pentagalloylglucose to induce oxidative stress in cancer cells was identified as a mechanistic event that led to or resulted from its cytotoxicity.

**Conclusions:**

We suggest that pentagalloylglucose is selectively cytotoxic to cancer cells, and at high concentrations could exhibit pro-oxidant effects in those cells, as opposed to its general anti-oxidant effects in cells. Also, the presence of **Compound 1** (pentagalloylglucose) in the plant and its cancer cell-selective cytotoxicity provide some rationale for the ethno-medicinal use of the plant’s leaf extract for treating diseases associated with excessive cell proliferation. Further studies are required to dissect the molecular mechanisms and players differentially regulating the biphasic anti-oxidant and pro-oxidant effects of pentagalloylglucose in normal and cancer cells.

## Background

Anacardiaceae is a family of plants well known for its tannin-rich constituents. The taxa and the vegetative parts being investigated determine the type of constituent tannin(s). Most members of Anacardiaceae are trees or shrubs located mainly in tropical but also in subtropical and temperate regions of the world, while the family is subdivided into 5 tribes, based on morphological characteristics [1–3], namely: Anacardiaceae, Rhoeae, Spondiadeae, Semecarparpaceae, and Dobineeae. The plants in these tribes have been widely explored as sources of many biologically useful polyphenols. Therefore, the presence of polyphenols in a member tribe is of chemotaxonomic significance. Chemotaxonomy seeks to utilize chemical information to improve the classification of plants [4]. However, beyond this, the presence of certain chemical constituents in a particular plant species could have a significant influence on the array of biological activities that could be obtained from such plant and, therefore, the medicinal uses for which it could be harnessed.

*Anacardium occidentale* L.*,* commonly referred to as Cashew, is a prominent member of the Anacardiaceae family. In Nigeria, it is one of the plants with multi-purpose medicinal uses. Almost all parts of the cashew tree have medicinal uses. The leaf is especially a common component of the recipes for many ailments. Reported ethno-medicinal uses of the leaf formulation include treatment of inflammation, infection, sore throat, asthma and oxidative conditions [5, 6]. It is also used in the treatment of fevers [7–9], aches and pains [8, 10], inflammation of the extremities [11], and asthma [12]. Mustapha [13] indicated the use of the leaf extract of *A. occidentale* in the treatment of Human immunodeficiency virus/ Acquired immunodeficiency syndrome (HIV/AIDS) opportunistic infections in the Northern part of Nigeria. *A.* o*ccidentale* was shown to possess anti-inflammatory effects in some models of inflammation [14]. A leaf extract of the plant showed anti-inflammatory and analgesic effects in the carrageenan-induced rat paw oedema and acetic acid-induced writhing assays, respectively [15]. Towards explaining the rationale for the application of the leaf of this plant for the treatment of cancerous diseases in ethno-medicine, this study isolated, from the plant part for the first time, pentagalloylglucose, a polyphenol with multiple medicinal applications. The cancer cell-selective cytotoxicity of pentagalloylglucose was demonstrated at its high concentrations to be partly as a result of, or causal to, the induction of oxidative stress.

## Methods

### Plant collection and preparation

Leaves of *A. occidentale* were collected at Road 9 Junction of the Senior Staff Quarters, Obafemi Awolowo University, Nigeria, in July 2015. The vegetative part was identified by Mr. Ogunlowo A.A. of the Department of Pharmacognosy, Faculty of Pharmacy, Obafemi Awolowo University, Ile Ife, Nigeria, and a voucher specimen (Voucher no. FPI 2107) was deposited in the IFE Herbarium. The leaves were air-dried and milled to give 2.5 kg of powdered material. The powdered leaves were extracted with 96 % ethanol. The extract was filtered and concentrated *in-vacuo* at 40^0^C to give 289 g (11.56 % w/w) of the crude ethanolic extract. The crude extract was dissolved in 200 mL of water and partitioned successively between water and *n-*hexane (3 x 500 mL), water and ethyl acetate (8 x 250 mL) and water and *n-*butanol (4 x 200 mL) to give *n-*hexane (26.2 g), ethyl acetate (15.8 g), *n-*butanol (14.6 g) and aqueous (210.4 g) fractions. The ethyl acetate fraction (previously determined to be active against some cancer cell lines) (15.1 g) was dissolved in methanol and adsorbed on silica gel and allowed to dry. The dry powder was packed into a column of silica gel (30 x 3 cm) and eluted in descending mode with varied proportions of solvent of increasing polarity from 100% hexane to 100% ethyl acetate to 50% methanol. The fraction that was eluted with 100% ethyl acetate was further subjected to repeated fractionation on silica gel and Sephadex LH-20 to give **Compound 1** (0.188 g).

### Chromatography and spectroscopic analysis

^1^H and ^13^C Nuclear magnetic resonance (NMR) spectra (for both 1D and 2D experiments) were obtained on the Bruker AV400 (IconNMR) Spectrometer at 400 and 100 MHz, respectively, while the Liquid Chromatography Mass Spectroscopy (LCMS) analyses were carried out on an Agilent LCMS comprising a 1100 series LC/MSD Trap SL at the School of Chemistry and Physics of the University of KwaZulu-Natal in Pietermaritzburg, South Africa. Adsorption chromatography (open column) was carried out with Silica gel (ASTM 230–400 mesh, Merck). Size exclusion column chromatography was achieved on Sephadex LH-20 (Pharmacia) pre-swollen in a specified solvent before loading onto the column. The column eluate was analyzed by Thin Layer Chromatography (TLC) performed at room temperature using analytical silica gel 60 GF_254_ pre-coated aluminum backed plates (Merck, 0.25 mm thick). The resulting spots on TLC plates were visualized under Ultraviolet (UV) light (254 nm) and detected by the use of 1% vanillin/H_2_SO_4_.

### Cell culture

The HeLa cell (immortalized human cervical cell line) and the MRC-5 SV2 cell (human foetal lung fibroblast line transfected with the virus SV40) were used as models of cancers, while the MRC-5 cell (human foetal lung cell line) was used as a model of normal (non-cancerous) cells. [16]. They were cultured in Dulbecco’s Modified Eagle Medium (DMEM) (4.5 g/L D-glucose) supplemented with 10% Foetal Calf Serum (FCS), 1% L-Glutamine (2 mM) and 1% antibiotic-antimycotic solution (penicillin/streptomycin/amphotericin B) and maintained at 37°C in a humidified atmosphere of 5% CO_2_ and 95% air [16]. All cells were from the European Collection of Authenticated Cell Cultures (ECACC), Salisbury, UK

### Cell viability assay to determine toxicity of compound

**Compound 1** at concentrations ranging from 6.25 to 100 μg/ml was evaluated for the potential to alter the viability of HeLa, MRC-5 SV2 and MRC-5 cells. Cells were seeded into opaque, flat bottom, microclear 96-well plates at 7.5 x 10^5^ cells/ml (7.5x10^4^ cells per well at 100 μl/well) and incubated for 24 h at 37°C and 5% CO_2_ to allow the cells to attach. After 24 h, the medium was discarded and the wells treated with 100 μl of the different concentrations of extracts prepared in growth medium. A set of untreated (negative) control wells was included in each plate as well as cells treated with doxorubicin (positive control) at concentrations between 0.1 and 20 μM.

Following incubation for up to 48 hours, 10 μl of the viability reagent MTT (3-(4,5-dimethylthiazol-2-yl)-2,5-diphenyltetrazolium bromide; 5 mg/ml in Phosphate-Buffered Saline (PBS)) was added to each well. After 2 hours of incubation with MTT at 37°C, the medium was discarded and 100 μl of DMSO was added to each well to dissolve the insoluble formazan formed. The absorbance at 570 nm was then determined with a microplate reader (CLARIO Star Microplate reader, BMG Labtech, UK) [16]. Each experiment was run in triplicate and repeated three independent times.

### Bright-field imaging to assess morphological damage

In order to assess treatment-induced changes to the morphology of the cells, bright-field images were acquired on an Olympus CKX41 microscope fitted with an Olympus DP71 U-TVIX-2 camera, using the Olympus cellSens entry software [16].

### Reactive Oxygen Species (ROS) Assay (DCFDA Assay)

HeLa cells were seeded into dark, clear-bottom 96-well microplates at 2.5 x 10^6^ cells/ml (2.5x10^5^ cells per well). The cells were incubated at 37°C and allowed to adhere overnight. The medium was thereafter aspirated from each well, followed by rinsing with 1X buffer provided in the assay kit (Abcam, Cat. No. ab113851). The buffer was aspirated and the cells stained with 100 μl of diluted 2′,7′-dichlorofluorescein diacetate (DCFDA) solution (25 μM). Stained cells were incubated for 45 min at 37°C in the dark. After 45 min, DCFDA solution was removed, cells were rinsed with 1x buffer, the rinse buffer was removed and the cells were treated, in duplicate, with 100 μl of **Compound 1** (6.25 to 100 μg/ml). The Fluorescence Intensity (FI) (Ex/Em = 485/535 nm) of each well was then read (CLARIO Star Microplate reader, BMG Labtech, UK) at 3 and 18 h following treatment. Background wells (untreated or diluent-treated stained cells), as well as blank wells (medium only), were included in each experiment. Each experiment was repeated three times. Cellular ROS data were then analysed and presented as fold changes compared to the negative control.

### Data presentation and analyses

For the viability assay data, the average viability of the negative control culture was taken as 100% and the average viability for every treatment was normalised to it. Values are indicated as Mean ± SEM (standard error of the mean). Statistical analyses were conducted with the GraphPad Prism Software (Version 8.0.1) (GraphPad Software Inc., CA, USA). To assess statistically significant differences between means, analysis of variance (ANOVA) was used, followed by a *post-hoc* test for multiple comparisons (Tukey test), with a p<0.05 considered statistically significant.

The IC_50_ value for each compound was calculated using GraphPad Prism (non-linear regression). Selectivity Index (SI) for **Compound 1** or doxorubicin was calculated by dividing the IC_50_ for its cytotoxic effect in the normal cell (MRC5) by the IC_50_ for its cytotoxic effect in the cancer variant (MRC5-SV2).

## Results

### Spectroscopic data

**Compound 1. IR (cm**^**-1**^**)** 3324, 1698, 1608. ^**1**^**H NMR (400 MHz, CD**_**3**_**OD)**. δH: 7.14 (2H, s, H-2′″/6′″), 7.09 (2H, s, H-2′/6′), 7.01 (2H, s, H-2″″/6″″ ), 6.98 (2H, s, H-2″″′/6′″″), 6.94 (2H, s, H-2′′/6′′), 6.26 (1H, d, *J=*8.0 Hz, H-1 ), 5.90(1H, m, H-4), 5.65 (1H, m, H-5), 5.61 (1H, m, H-2), 4.42 (1H, m, H-3), 4.39 (2H, m, H-6). ^**13**^**C NMR (100 MHz, CD**_**3**_**OD).** δc : 93.8 (CH, C-1), 74.1 (CH, C-3), 72.2 (CH, C-4), 70.8 (CH, C-2), 68.4 (CH, C-5), 62.2 (CH_2_, C-6). Galloyl i: 119.7 (CH, C-1′), 110.3 (CH, C-2′/6′), 140.0 (CH, C-4′), 146.2 (CH, C-3′/5′), 166.2 (C=O, C-7′), Galloyl ii: 120.2 (CH, C-1″), 110.3 (CH, C-2″/6″), 140.1 (CH, C-4″)146.4 (CH, C-3″/5″), 166.9 (C=O, C-7″); Galloyl iii; 121.1 (CH, C-1″′), 110.7 (CH, C-2″′/6″′), 146.5 (CH, C-3″′/5″′), 140.8 (CH, C-4″′), 167.9 (C=O, C-7″′); Galloyl iv; 120.2 (CH, C-1″′′), 110.4 (CH, C-2″′′/6″′′), 146.4 (CH, C-3″′′/5″′′), 140.3 (CH, C-4″′′), 167.0 (C=O, C-7″′′). Galloyl v: 120.2 (CH, C-1″′′′), 110.4 (CH, C-2″′′′/6″′′′), 146.4 (CH, C-3″′′′/5″′′′), 140.3 (CH, C-4″′′′), 167.0 (C=O, C-7″′′′). **TOF HRMS** ***m/z*** 963.1135 [M+Na]^+^ (calculated 963.1080).

### Structural elucidation of the isolated compound

**Fig. 1:**
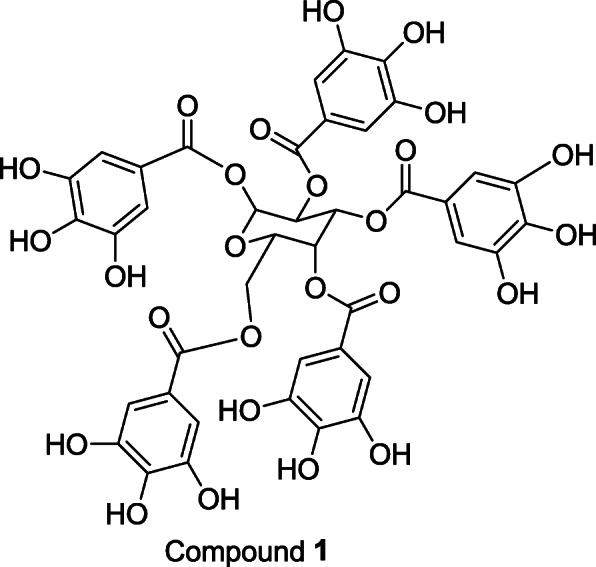
Structure of isolated **Compound 1**

**Compound 1** was isolated as a brown amorphous powder. The compound gave a strong blue-black colour on the thin layer chromatography when sprayed with ferric chloride, indicating the presence of phenolic moiety. IR spectrum displayed absorption at 3324, 1698, 1608, 1536 cm^-1^. Positive Time-of-Flight High Resolution Mass Spectrometry (TOF HRMS) gave a signal at *m/z* 963.1135 [M+Na]^+^ (calculated 963.1080) for a molecular mass C_41_H_32_O_26_. The proton NMR spectrum displayed five singlets at δH 7.14, 7.09, 7.01, 6.98, and 6.94, characteristic of multiply substituted gallotannins. The appearance of a doublet at 6.26 ppm with a large coupling constant (*J=* 8.4 Hz) indicates the presence of a β-anomeric proton. The sugar protons were assigned based on the COSY and HMBC spectra. In the COSY spectrum, these pairs of correlating protons were observed between the signals at δH 6.26 / 5.61; 5.61 /6.26, 4.42, 5.90 /5.65, while in the HMBC spectrum long range correlations were observed between the protons at δH 6.26 and δc 72.9, 164.7; δH 4.42 and δc 69.5, 166; δH 5.90 and δc 62.1, 73.0, 92.6 and 166. Each galloyl group was placed based on the correlation between the sugar protons as well as the aryl singlet protons with the galloyl carbonyl carbons. **Compound 1** was identified as β-penta-*O-*galloyl glucose (PGG) (molecular weight: 940.68) (Figure 1) by comparison of the spectroscopic data with literature values [17].

### Cytotoxicity of the isolated compound

**Fig. 2:**
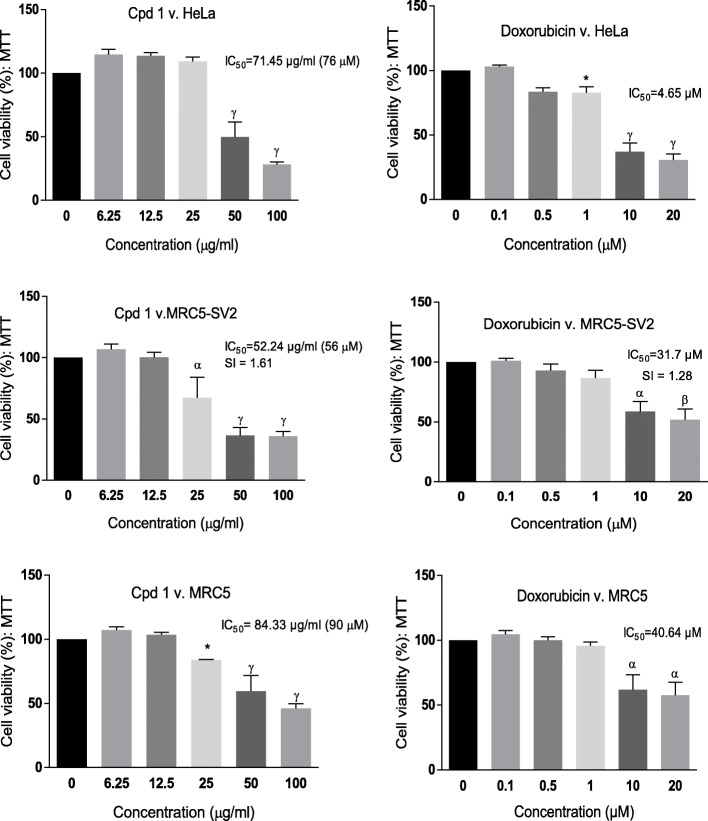
Cell viability (%) of HeLa, MRC5-SV2 and MRC5 cells following 24 h exposure to **Compound 1** (Cpd 1) and doxorubicin. Each bar represents mean ± SEM (n=3); *p<0.05, ^α^p<0.01, ^β^p<0.001, ^γ^p<0.0001 vs. negative control using one-way ANOVA followed by Dunnett’s *post hoc* multiple-comparison test. SI is Selectivity Index.

**Compound 1** showed concentration-dependent toxicity to HeLa, MRC5 and MRC5-SV2 cells following 24 h exposure to varying concentrations up to 100 μg/ml (Figure 2). The IC_50_ obtained for **Compound 1** was 71.45 μg/ml (76 μM), 52.24 μg/ml (56 μM) and 84.33 μg/ml (90 μM) in HeLa, MRC5-SV2 and MRC5 cells respectively; the selectivity index for **Compound 1**, which is the ratio of its IC_50_ in MRC5 and MRC5-SV2 cells, was calculated as 1.61. Doxorubicin, used as a standard anti-cancer drug, also demonstrated concentration-dependent toxicity to HeLa, MRC5 and MRC5-SV2 cells following 24 h exposure to it (Figure 2), with IC_50_ of 4.65, 31.70 and 40.64 μM in HeLa, MRC5-SV2 and MRC5 cells respectively. The selectivity index for doxorubicin was 1.28.

**Fig. 3:**
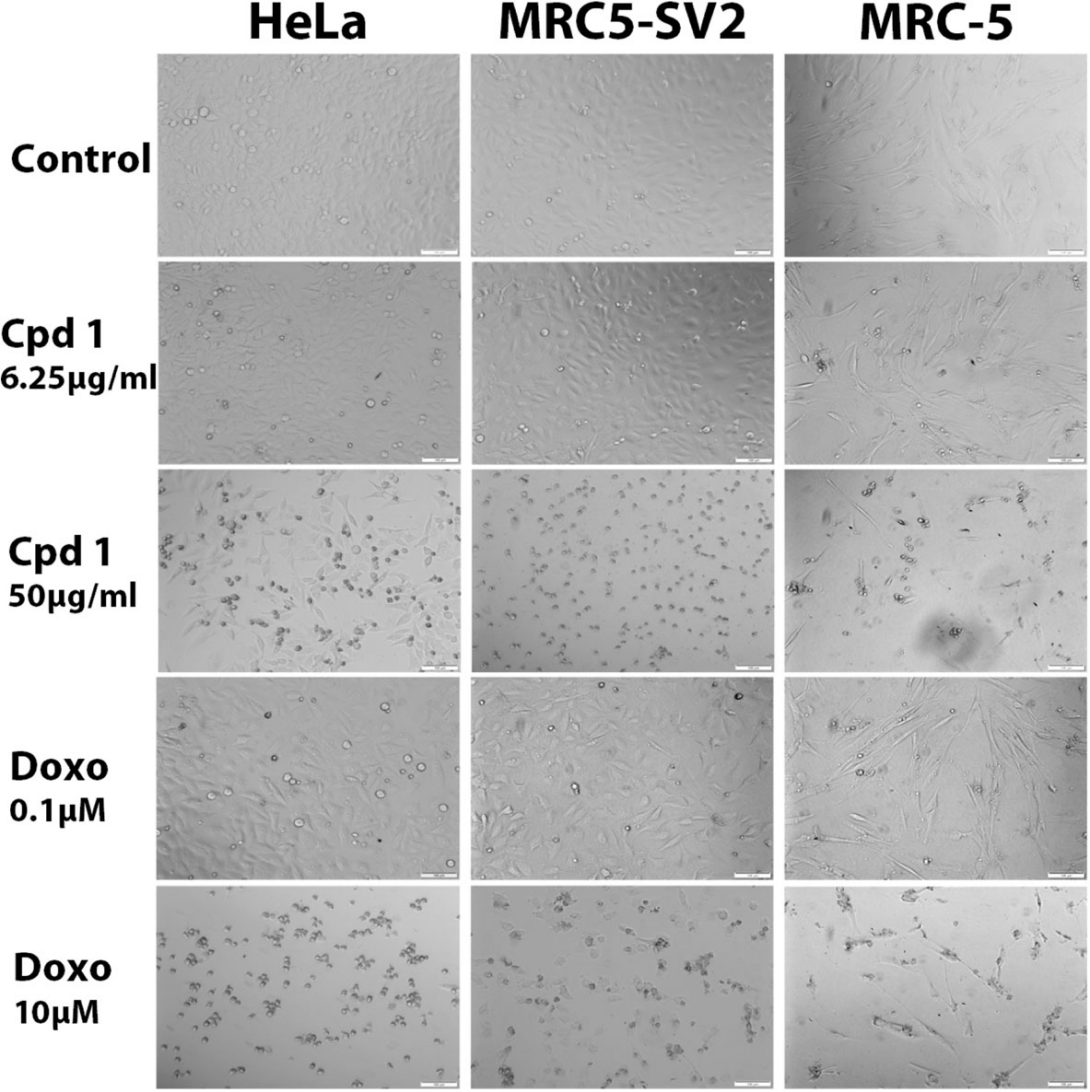
Concentration-dependent damaging effects of **Compound 1** (Cpd 1) and doxorubicin (doxo) on the morphology of HeLa, MRC5-SV2 and MRC5 cells following exposure of the cells to either compound for 24 h. Scale bar = 100 μm.

The concentration-dependent toxicity of **Compound 1** and that of the positive control doxorubicin were correlated with morphological damage that worsened as the concentration of each compound increased. As shown in Figure 3 for the HeLa, MRC5-SV2 and MRC5 cells, a higher concentration of **Compound 1** or doxorubicin caused loss of cells and rounding up of several or nearly all remaining cells, while control cells not exposed to either compound were confluent and their connections were intact.

While the MRC5-SV2 cell was more sensitive to **Compound 1** than the HeLa cell, it was nearly seven times less sensitive to doxorubicin than the HeLa cell. **Compound 1** also showed better selectivity for cancer cells (as against normal cells) than doxorubicin. We thus decided to explore in a cancer cell aspects of the time course of its induction of cytotoxicity by comparing its effects with those of doxorubicin, following treatment with each compound at the same range of concentrations as was tested before but for much shorter durations of 3 h and 6 h. This was assessed using the HeLa cell that was less sensitive to **Compound 1** than doxorubicin.

**Fig. 4:**
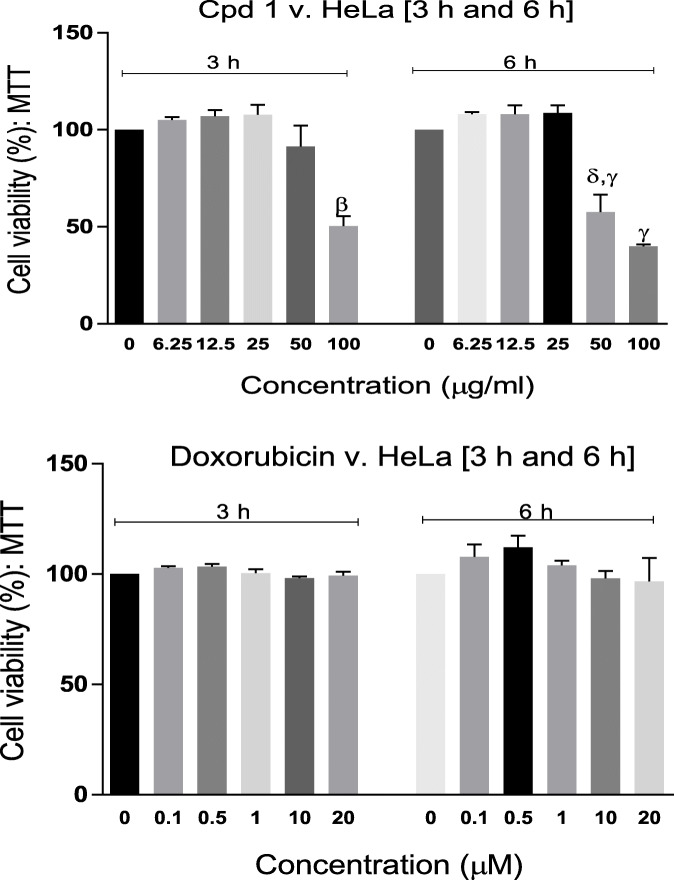
Cell viability (%) of HeLa cells following 3 h and 6 h exposures to **Compound 1** (Cpd 1) and doxorubicin**.** Each bar represents mean ± SEM (n=3); ^β^p<0.001, ^γ^p<0.0001 vs control, ^δ^p<0.01 vs corresponding 3 h observation using one-way ANOVA followed by Dunnett’s *post hoc* multiple-comparison test.

As shown in Figure 4, following 3 h and 6 h exposure of HeLa to **Compound 1** and doxorubicin, **Compound 1** demonstrated concentration- and time-dependent toxicity, which was significant at 50 and 100 μg/ml, while doxorubicin showed no toxicity at the two time points. This observation establishes a key difference in the cytotoxicity time-course profiles of **Compound 1** and doxorubicin. While **Compound 1** rapidly induced cytotoxicity, initiated from as early as 3 h following exposure of cells to it and progressively increasing up to 24 h, doxorubicin’s toxicity revealed a much-slower time-course, with significant, concentration-dependent toxicity only observed after 24 h exposure to it. For drug discovery and development purposes, this property of **Compound 1** could make it uniquely promising, as a shorter time of exposure to an anti-cancer agent could ensure less damage to normal cells and less side effects.

### Induction of reactive oxygen species (ROS) as a potential mechanism by which compound elicits cytotoxicity

**Fig. 5:**
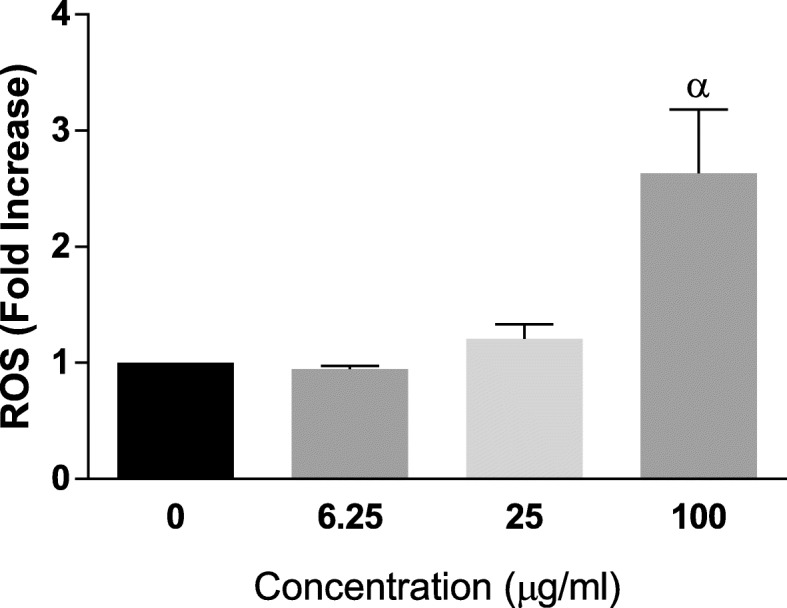
Concentration-dependent increases in intracellular Reactive Oxygen Species (ROS) elicited by **Compound 1** in HeLa cells, presented as fold increases compared to the negative control, following 3 h exposure of HeLa cells to **Compound 1**. Each bar represents Mean ± SEM (n=3); ^α^p<0.01 versus control using one-way ANOVA followed by Dunnett’s *post hoc* multiple-comparisons test.

As we established **Compound 1** as rapidly and selectively cytotoxic to cancer cells, we assessed whether the generation of ROS was a mechanism by which it induced its toxicity in cancer cells, at least, in part. Figure 5 reveals that, at 3 h post-treatment in HeLa cells, **Compound 1** up to 25 μg/ml did not induce significant ROS but at 100 μg/ml caused a significant increase in ROS levels. This almost 3-fold increase in ROS could be correlated with an almost 50% decrease in viability at the 3 h time point (cf. Figure 4, top panel, left hand side - for 3 h).

## Discussion

We have previously reported the cytotoxicity of the crude extract of the leaf of ***Anacardium occidentale*** [18]. The results from the current study demonstrate the cancer cell-selective cytotoxicity of pentagalloylglucose obtained from the leaf extract. At high concentrations, the observed cytotoxicity of pentagalloylglucose is partly as a result of, or causal to, the induction of oxidative stress. Reactive oxygen species (ROS) have been implicated in DNA mutations, aging, and cell death [19]. While low-to-moderate ROS levels can be beneficial to normal cells by promoting proliferation pathways, high ROS levels can be detrimental to normal and tumour cells and induce cell death [20]. Some antineoplastic drugs that are currently used for cancer chemotherapy (e.g., doxorubicin, taxanes, vinca alkaloids, and antimetabolites) induce high levels of oxidative stress [21, 22], by which they kill cancer cells.

It should be noted that pentagalloylglucose has been generally reported to be an antioxidant, with some reports establishing it as a potent antioxidant [23–26] and potentially useful in chemoprevention [27], thus suggesting it should be relatively non-cytotoxic, which might appear to contrast with observations of its cytotoxicity and, at high concentrations, its pro-oxidant effect (induction of oxidative stress) in cancer cells reported in this paper. However, consistent with our findings, previous studies undertaken using cell cultures, as we did, have shown that pentagalloylglucose could be involved in eliciting anti-cancer effects through mechanisms including pro-apoptosis, anti-proliferation, anti-angiogenesis, anti-metastasis and inhibition of glycoprotein [28]. Besides, Kantapan et al. [29] recently reported that extracts containing pentagalloylglucose could promote intracellular ROS production and induce apoptosis in cancer cells, although it was not demonstrated whether those effects could be solely or partly attributed to the presence of pentagalloylglucose, as two other constituents were found to be present in the extract. Lin et al. [30], who used cultured cells (HepG2, 293T, HEp-2, MRC-5) grown and treated in conditions very similar to ours (DMEM supplemented with 10% fetal calf serum, 24 h treatment, MTT assay), showed pentagalloylglucose lacked toxicity to the cells up to 50 μM but did not investigate higher concentrations.

It is generally recognised that some antioxidant molecules are capable of exhibiting an antioxidant-pro-oxidant switch, depending on a number of factors, including the redox state of the cellular environment or the nature of the pathology in question. We reckon that, while pentagalloylglucose is generally antioxidant in nature, it could, under certain conditions, especially in cancer cells and at high concentrations, generate ROS, consistent with a pro-oxidant effect, and induce cytotoxicity. While our work showed that pentagalloylglucose concentrations above 25 μM but below 100 μM could induce rapid cytotoxicity in cancer cells, there was no evidence that the cytotoxicity involved significant ROS, and it is thus reasonable to consider it to be independent of ROS, meaning other toxic mechanisms were involved. However, at high concentrations (100 μM and above), the toxicity is suggested to result from, or cause, substantial ROS. Overall, we suggest a dynamic and complex scenario in which cell toxicity, where it occurs, is independent of, results from, causes, or is synergistic with, elevated ROS levels.

Taken together, the data show that **Compound 1**, identified as β-penta-O-galloyl glucose, could induce rapid and selective cytotoxicity in cancer cells, which at high concentrations could be linked to oxidative stress. Further studies will identify the molecular mechanisms and players responsible for the anti-oxidant and pro-oxidant effects of pentagalloylglucose in normal and cancer cells.

## Conclusions

This study isolated pentagalloyl glucose, a functionally valuable gallotannin from the leaf extract of *Anacardium occidentale* for the first time. Our results show that pentagalloylglucose is more toxic to cancer cells than to normal cells, produces rapid decreases in tumour cell populations, and at higher concentrations generates ROS, leading to oxidative stress in cancer cells. The presence of the compound pentagalloylglucose in the plant species is of chemotaxonomic significance and partly validates the use of the leaf extract of *Anacardium occidentale* in ethno-medicine for treating cancers and related pathologies.

## Data Availability

Data sets have not been deposited in any repository but are available from corresponding authors upon request

## Abbreviations

DCFDA: 2′,7′-dichlorofluorescin diacetate
DMEM: Dulbecco’s Modified Eagle Medium
DMSO: Dimethyl sulfoxide
DNA: Deoxyribonucleic acid
FCS: Foetal Calf Serum
HIV/AIDS: Human immunodeficiency virus/ Acquired immunodeficiency syndrome
HRMS: High-resolution mass spectrometry
IR: Infrared Spectroscopy
LCMS: Liquid Chromatography Mass Spectrometry
MTT: 3-(4,5-dimethylthiazol-2-yl)-2,5-diphenyltetrazolium bromide
NMR: Nuclear magnetic resonance
ROS: Reactive Oxygen Species
SEM: Standard Error of Mean
SI: Selectivity Index
TLC: Thin Layer Chromatography
TOF: Time-of-Flight
UV: Ultraviolet
